# Neuregulin and BDNF Induce a Switch to NMDA Receptor-Dependent Myelination by Oligodendrocytes

**DOI:** 10.1371/journal.pbio.1001743

**Published:** 2013-12-31

**Authors:** Iben Lundgaard, Aryna Luzhynskaya, John H. Stockley, Zhen Wang, Kimberley A. Evans, Matthew Swire, Katrin Volbracht, Hélène O. B. Gautier, Robin J. M. Franklin, Charles ffrench-Constant, David Attwell, Ragnhildur T. Káradóttir

**Affiliations:** 1 Wellcome Trust–Medical Research Council (MRC) Stem Cell Institute, John van Geest Centre for Brain Repair, and Department of Veterinary Medicine, University of Cambridge, Cambridge, United Kingdom; 2 Department of Pathology, University of Cambridge, Cambridge, United Kingdom; 3 MRC Centre for Regenerative Medicine, Centre for Multiple Sclerosis Research, University of Edinburgh, Edinburgh, United Kingdom; 4 Department of Neuroscience, Physiology & Pharmacology, University College London, London, United Kingdom; Stanford University School of Medicine, United States of America

## Abstract

Myelination is essential for rapid impulse conduction in the CNS, but what determines whether an individual axon becomes myelinated remains unknown. Here we show, using a myelinating coculture system, that there are two distinct modes of myelination, one that is independent of neuronal activity and glutamate release and another that depends on neuronal action potentials releasing glutamate to activate NMDA receptors on oligodendrocyte lineage cells. Neuregulin switches oligodendrocytes from the activity-independent to the activity-dependent mode of myelination by increasing NMDA receptor currents in oligodendrocyte lineage cells 6-fold. With neuregulin present myelination is accelerated and increased, and NMDA receptor block reduces myelination to far below its level without neuregulin. Thus, a neuregulin-controlled switch enhances the myelination of active axons. *In vivo*, we demonstrate that remyelination after white matter damage is NMDA receptor-dependent. These data resolve controversies over the signalling regulating myelination and suggest novel roles for neuregulin in schizophrenia and in remyelination after white matter damage.

## Abstract 2

Neuregulin switches oligodendrocytes between two modes of myelination: from a neuronal activity–independent mode to a myelin-increasing, neuronal activity–dependent, mechanism that involves glutamate release and NMDA receptor activation.

## Author Summary

Myelination acts as an insulator for neurons and as such is essential for normal brain function, ensuring fast neuronal communication. Oligodendrocytes are the cells that wrap their membrane around nerve cell axons to form the myelin sheath that enables fast action potential propagation. However, what determines whether an individual axon becomes myelinated remains unknown. We show that there are two distinct modes of myelination: one that is independent of neuronal activity and the release of the neurotransmitter glutamate and another that depends on nerve cell action potentials releasing glutamate, which then activates a class of glutamate receptor (NMDA receptors) on oligodendrocyte lineage cells. We find that the protein neuregulin switches oligodendrocytes between these two modes of myelination; neuregulin increases oligodendrocyte lineage cells' sensitivity to glutamate by increasing the current flowing through their glutamate receptors. With neuregulin present, myelination is accelerated and increased. Blocking NMDA receptors reduces the amount of myelination to far below its level without neuregulin. Thus, a neuregulin-controlled switch enhances the myelination of active axons. We also demonstrate that remyelination after white matter damage (as occurs in diseases, such as spinal cord injury and multiple sclerosis) is NMDA receptor-dependent. These data help us understand the signalling that regulates myelination and suggest the possible involvement of neuregulin in schizophrenia and in remyelination after white matter damage.

## Introduction

Myelination is essential for normal brain function as myelin sheaths provide trophic support for axons and increase the conduction speed of action potentials [1]. By speeding axonal action potentials, myelin allows rapid information transfer between different regions of the CNS. Consequently, the increased amount of white matter in human brains is thought to contribute significantly to our cognitive powers. Myelination is controlled by a complex set of factors, including growth factors, interactions between axons and their myelinating glia, and downstream signalling processes within the glia [2]. Understanding the myelination process is crucial, not only to understand in full how action potential speed can be increased to enhance the brain's cognitive power, but also to develop therapeutic strategies for regenerating myelin in disease. Strikingly, however, we still do not know what determines whether an individual axon becomes myelinated. In particular, for the CNS, the roles of the growth factor neuregulin (NRG), neuronal activity, and neurotransmitter glutamate release from axons are highly debated.

NRG on axons can regulate myelination by signalling to ErbB receptors on ensheathing Schwann cells or oligodendrocytes, but there is controversy over its actions. In the peripheral nervous system, a decrease of NRG signalling leads to less myelination [3],[4]. Decreasing NRG-ErbB signalling has also been reported to reduce myelination by oligodendrocytes in the central nervous system [5]–[8]. Contradicting this, however, knocking out NRG or ErbB was found to have no effect on myelination, although overexpressing NRG increased myelination [9].

Further uncertainty relates to how neuronal activity regulates CNS myelination. Neuronal activity can promote myelination [10],[11], yet oligodendrocytes can ensheath dead axons that lack any activity [12]. An activity dependence to myelination could reflect action potential evoked release of NRG [13], but glutamate is also released onto oligodendrocyte precursor cells by action potentials in unmyelinated axons [14]–[17] and could potentially initiate myelination. Glutamate activates both AMPA/kainate and NMDA receptors in oligodendrocyte lineage cells [14]–[18], and the presence of NMDA receptors in oligodendrocyte processes [18]–[20] is consistent with them having a role in myelination. Furthermore, glutamate signalling and NRG signalling might interact to control myelination, since, in the grey matter at least, NRG increases the expression of NMDA receptor subunits in neurons [21] and in forebrain [22]. However, contradictory data have been presented on the contribution of NMDA receptors to CNS myelination, with suggestions that they either play no role [23],[24] or that their activation by glutamate released from axons promotes myelin basic protein (MBP) expression and myelination [16],[25].

Here we show for the first time that there are two distinct modes of myelination by oligodendrocytes, one independent of neuronal activity and the other dependent on neuronal action potentials. Furthermore, we demonstrate that NRG switches oligodendrocytes between these two myelination programmes by increasing NMDA receptor-mediated currents in oligodendrocyte lineage cells, making these cells more sensitive to glutamate released from active axons. As a result, this interaction of NRG and glutamatergic signalling accelerates and increases myelination, but most importantly provides a mechanism by which myelination is focussed on the axons of active neurons. Investigations of white matter pathology based on this finding revealed that remyelination of axons in a demyelinated lesion *in vivo* is NMDA receptor dependent, suggesting enhancement of NRG- and NMDAR-dependent remyelination as a therapeutic approach for promoting recovery after demyelination.

## Results

### NRG Increases Myelination by Oligodendrocytes

**Figure 1:**
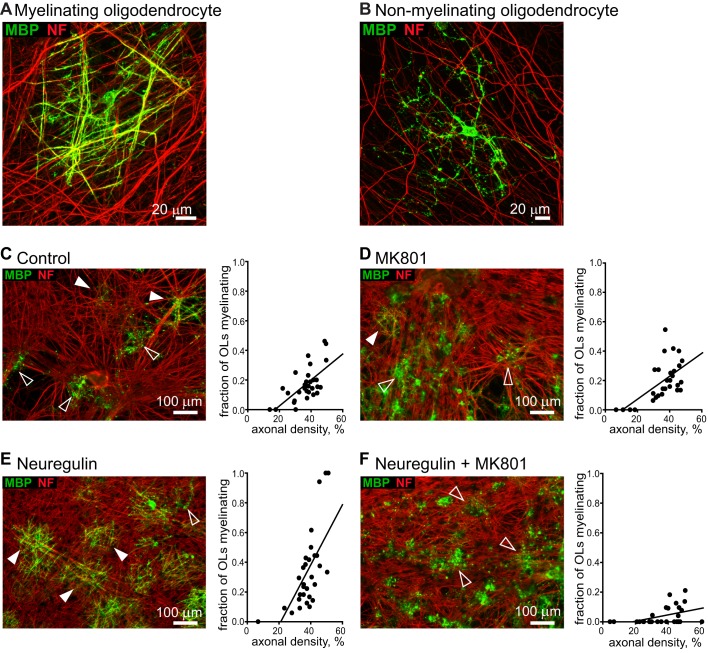
Effect of NRG and NMDA receptor block on myelination. (A, B) High-magnification views of a myelinating oligodendrocyte (A) with MBP (green) expressed in processes wrapping around axons expressing NF 160/200 (NF, red), and of a nonmyelinating oligodendrocyte (B) with MBP expressed (in a more patchy and often diffuse manner) in processes that are not aligned with axons. Myelination was quantified as the fraction of all MBP-expressing oligodendrocytes that provided a thick straight myelin sheath to at least one axon. (C–F) Myelinating processes (MBP, green) wrapping DRG axons (NF, red) in control conditions (C), in the presence of MK-801 (D), in the presence of NRG (E), and in the presence of NRG and MK-801 (F). Filled and open arrows show some myelinating and nonmyelinating oligodendrocytes. Graphs show fraction of oligodendrocytes that are myelinating, versus fraction of area occupied by DRG processes, for 30 images of each coverslip from which the specimen images shown were taken, best fit with a linear dependence of myelination on axon density.

To assess whether NRG-ErbB signalling and activation of NMDA receptors in oligodendrocytes interact to control myelination, we studied myelination of cultured dorsal root ganglion (DRG) neurons by forebrain oligodendrocytes [8]. This allows more detailed investigation of the underlying signalling mechanisms than is possible in transgenic studies where compensation for gene knockout may occur (see Discussion). In this coculture system, compact myelin is produced (Figure S1A) [8] with a lamellar repeat distance of 10.9±1.8 nm (*n* = 6) and a g-ratio of 0.74±0.02 (*n* = 6), as expected for CNS myelination [26],[27]. Myelinating and nonmyelinating oligodendrocytes are distinguishable after labelling for MBP and neurofilament (NF) (Figure 1A,B). Myelinating oligodendrocytes enwrap NF-expressing axons with MBP-expressing processes, at the lateral ends of which the paranode labels for the axon-oligodendrocyte junction protein Caspr (Figure S1B). When two oligodendrocytes myelinate adjacent segments of the same axon, a node of Ranvier is identifiable by the presence of Caspr labelling flanked by MBP labelling on either side of the node (Figure S1C). Myelination does not occur in the absence of added OPCs, it is mediated purely by OPCs and not by Schwann cells, and only DRG neurons and not interneurons become myelinated (Figure S1D–F).

**Figure 2:**
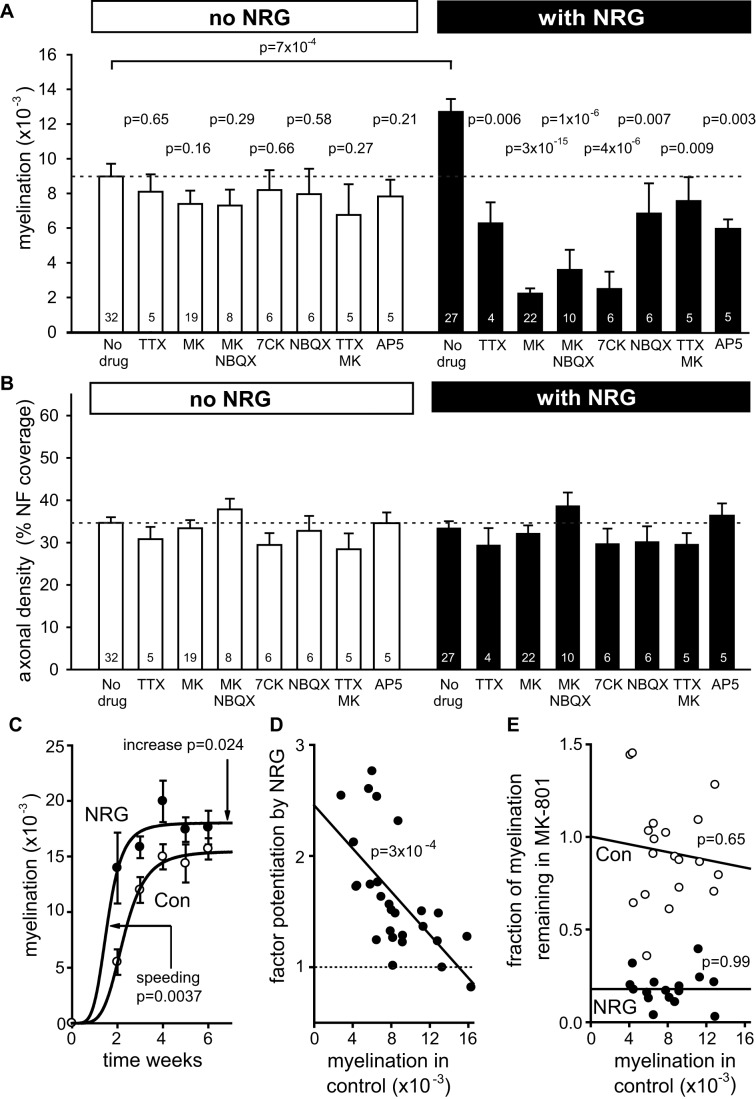
NRG switches myelination to an activity- and NMDA receptor-dependent programme. (A) Mean myelination parameter (the value of A from eqn. 1 of the Materials and Methods) from experiments as in Figure 1 for different conditions (number of experiments shown on bars). For *No NRG*, ANOVA indicated no significant differences across all bars (*p* = 0.79); *p* values from *t* tests are for comparison with control. For *With NRG*, ANOVA showed significant differences across all bars (*p*<0.0001); *p* values (from Holm–Bonferroni post hoc test) are for comparison with NRG alone (comparison between conditions with and without NRG: TTX versus NRG+TTX *p* = 0.29; MK versus NRG+MK *p* = 6×10^−7^; MK+NBQX versus NRG+MK+NBQX *p* = 0.14; 7CK versus NRG+7CK *p* = 0.02; NBQX versus NRG+NBQX *p* = 0.65; TTX+MK versus NRG+TTX+MK *p* = 0.72; AP5 versus NRG+AP5 *p* = 0.85). (B) Axon density (fraction of image pixels labelled for NF 160/200) for the conditions in (A) (ANOVA showed no significant differences, *p* = 0.19). (C) Myelination in control and NRG at different times after plating OPCs onto DRG cells (5–7 cocultures per point). Plots are myelination (M) as a function of time (t) where M = M_max_.t^n^/(t^n^+T^n^) with n fixed at 4.7 (best fit for control) and best fit values were M_max_ = 0.0180 (NRG) or 0.0155 (Con) and T = 1.56 (NRG) or 2.28 (Con) weeks. The *p* values are shown for the increase of M_max_ and decrease of T in NRG compared to control. (D) Potentiation of myelination by NRG as a function of the level of myelination in control conditions (each point is one individual coculture). (E) Fraction of myelination remaining in MK-801 as a function of the level of myelination in control conditions (each point is one individual coculture; variability in the data reflects taking the ratio of two variable levels of myelination), in the absence (Con) and presence of NRG. See also Figure S2 and S3.

ErbB signalling was activated by applying the extracellular domain of NRG (NRG1 type 1-β1, 10 ng/ml, 0.33 nM), which includes the EGF domain that binds to ErbB receptors expressed on oligodendrocytes [28] and mimics [3] the effect of membrane-bound NRG. As found previously [8], adding NRG increased the fraction of oligodendrocytes that myelinated axons (Figure 1C,E, Figure 2A). Myelination depends on the local axon density [8], so we quantified myelination (3 wk after plating OPCs on the neurons) as the slope of a plot of the fraction of oligodendrocytes that were myelinating against local axon density [8] (see Figure 1C–F, Figure 2A, and Materials and Methods; similar results were obtained with other quantification methods, see Figures S2 and S3). Measured in this way, adding NRG increased myelination by 42% (*p* = 7×10^−4^; Figure 2A). NRG also increased MBP protein expression in the cocultures (*p* = 0.03; Figure S1G), without affecting MBP or MOG mRNA levels (Figure S1H), indicating a local posttranscriptional regulation of these myelin genes [16].

Varying the time at which myelination was assessed after plating the OPCs onto the neurons showed that NRG both accelerated myelination and increased its steady state level (Figure 2C). The myelination at steady-state level was 16% higher (*p* = 0 .024), and its onset (defined as the time to reach half the maximal value) occurred 5 d earlier (*p* = 0.0037), according to the fitted curves in Figure 2C.

**Figure 3:**
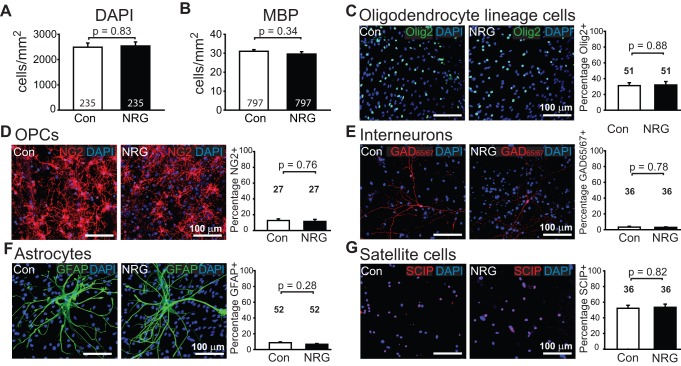
NRG has no effect on cell density. (A, B) Total number of DAPI-labelled cells (A) and number of MBP-expressing oligodendrocytes (B) in control and NRG-treated cultures. (C–G) Images of labelled cells in control and NRG, and bar chart showing the percentages of cells, which were (C) Olig2-expressing oligodendrocyte lineage cells, (D) NG2-expressing OPCs, (E) GAD-expressing interneurons, (F) GFAP-expressing astrocytes, and (G) SCIP-expressing satellite cells. Number of fields of view counted per condition are shown on bars; 10 fields were imaged per coverslip.

To assess whether changes in cell proliferation, growth, or survival were responsible for the increased myelination in NRG, we counted cells of different types identified with antibodies. In the presence of NRG, there was no significant change in the density of axons (Figure 2B), the total number of DAPI-labelled cells (Figure 3A), the number of MBP-labelled oligodendrocytes (Figure 3B), or the percentage of cells that were oligodendrocyte lineage cells, OPCs, interneurons, astrocytes, or satellite cells (Figure 3C–G) in the cocultures. No microglia were detected using anti-CD11b antibody or isolectin B4. Thus, the effect of NRG is not due to an altered cell density or changes in environmental paracrine signals or trophic factors caused by altered cell numbers.

### NRG's Effect Depends on Action Potentials

To test the role of neuronal activity in regulating myelination we applied TTX (1 µM) to block action potentials. TTX had no effect on myelination in the absence of added NRG (*p* = 0.65), but reduced myelination by 50% in the presence of NRG (*p* = 0.006; Figure 2A)—that is, TTX abolished the increase in myelination produced by NRG (both when added at the same time as NRG and when added 3 d later; Figure S5A). Thus, whereas in the absence of NRG myelination occurs predominantly by a programme that is independent of neuronal activity, with NRG present myelination depends partly on action potentials.

**Figure 4:**
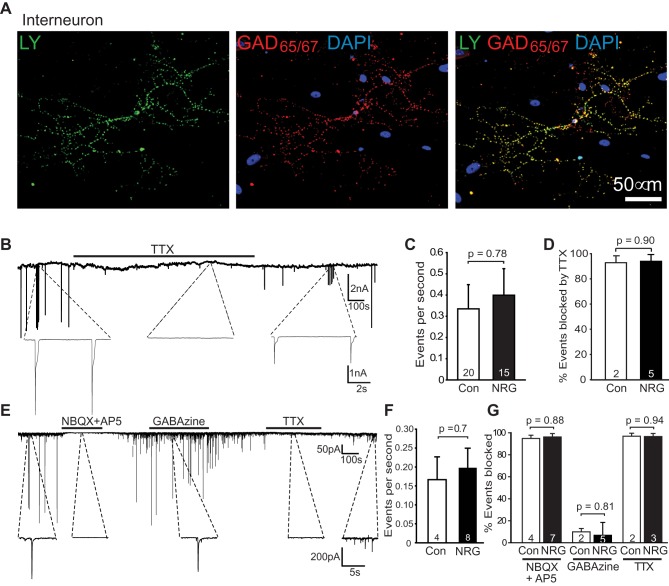
NRG does not affect neuronal activity. (A) Lucifer yellow filled patch-clamped interneuron, identified after recording by labelling for glutamate decarboxylase (GAD) 65/67, from which action potential evoked synaptic currents were recorded. DAPI labelling shows cell nuclei. (B) Spontaneous synaptic currents at −64 mV recorded in an interneuron with E_Cl_ = 0 mV. (C–D) Event frequency (C) and block by 1 µM TTX (D) in control and NRG cultures. (E) Spontaneous synaptic currents at −70 mV recorded with E_Cl_ = −88 mV, during application of 25 µM NBQX+50 µM D-AP5, or 10 µM GABAzine, or 1 µM TTX. (F–G) Event frequency (F) and block by NBQX/AP5, GABAzine, and TTX (G) in control and NRG cultures.

Because myelination can be promoted by neuronal activity [10],[11], we assessed whether NRG increased action potential activity in the DRG axons being myelinated in the cocultures, by voltage-clamping interneurons (Figure 4A) and measuring the frequency of synaptic input they received from DRG neurons (Figure 4B). NRG did not significantly affect the total frequency of (excitatory plus inhibitory) synaptic currents recorded at −64 mV with a high [Cl^−^]_pipette_ (Figure 4B,C), 94% of which were blocked by TTX (Figure 4D) and so reflect action potential activity in the cocultures. At −70 mV with E_Cl_ set to −88 mV so that all inward currents are EPSCs from DRG cells (Figure 4E), there was no difference in the frequency of events in control and NRG cultures (Figure 4F; 25 µM NBQX+50 µM AP5 blocked the currents, as did TTX, while 10 µM GABAzine had essentially no effect on them; Figure 4E,G). Thus, NRG makes myelination of DRG axons become dependent on neuronal activity without increasing the level of that activity.

### NRG Makes Myelination Become NMDA Receptor Dependent

These data suggest that NRG increases the oligodendrocyte sensitivity to events triggered by axonal action potentials. To investigate this idea, we tested the effects of glutamate receptor blockers. The NMDA receptor channel blocker MK-801 (10 µM) had no effect on myelination in the absence of added NRG (Figure 1C–D, Figure 2A; *p* = 0.16) but reduced myelination by 82% when NRG was present (Figure 1E–F, Figure 2A; *p* = 3×10^−15^). In NRG+MK-801, myelination was reduced to only 25% of the level seen in control conditions in the absence of NRG (*p* = 4×10^−9^). The same results were obtained (without and with NRG) when the AMPA/kainate receptor blocker NBQX (25 µM) was added with the MK-801 (Figure 2A). Thus, NRG induces a switch in the main mode of myelination, from being completely independent of NMDA receptor activation in the absence of NRG to being mainly (82%) dependent on NMDA receptor activation when NRG is present. Similarly, the structurally unrelated NMDA receptor blocker 7-chlorokynurenate (30 µM) also had no effect in the absence of NRG (*p* = 0.66) but reduced myelination in the presence of NRG by 80% (*p* = 4×10^−6^), that is, to only 28% of the control level with no NRG (*p* = 0.002; Figure 2A). The blocker of the glutamate site on NMDA receptors, D-AP5 (200 µM) reduced myelination in NRG less than MK-801 or 7-chlorokynurenate (Figure 2A) for reasons discussed below. NBQX alone had no effect on myelination (*p* = 0.58) in the absence of NRG, but reduced myelination by 46% in NRG (*p* = 0.007; Figure 2A). Thus, the activity-dependent myelination induced by NRG depends on activation of NMDA and AMPA/kainate receptors.

To assess how the effects of NRG and NMDA receptor block varied with the initial level of myelination, we took advantage of variations between cocultures in the control level of myelination. When myelination was low in control conditions the potentiation by NRG was large (reaching an extrapolated value of 2.5±0.2-fold at zero myelination), but the NRG effect became much smaller when the control level of myelination was large (Figure 2D). Strikingly, however, despite this variation in NRG's potentiating action, the 5-fold reduction of myelination produced in NRG by blocking NMDA receptors with MK-801 occurred independently of the control level of myelination (Figure 2E). Thus, NRG makes myelination become highly dependent on the activation of NMDA receptors even when the increase in myelination produced by NRG is small.

### NRG Increases NMDA Receptor Currents in Oligodendrocyte Lineage Cells

**Figure 5:**
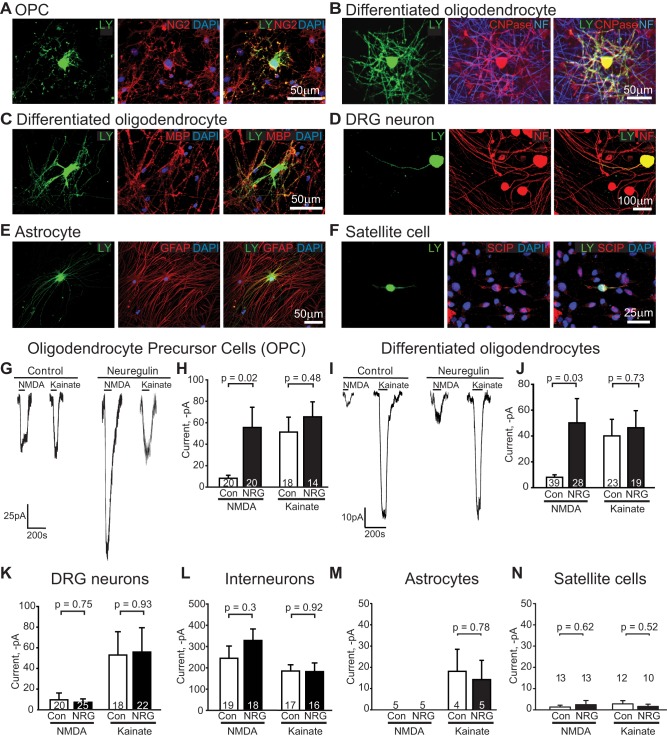
NRG specifically increases NMDA receptor currents in oligodendrocyte lineage cells. (A–F) Images of Lucifer yellow in patch-clamped cells (left), antibody labelling for identification (middle), and merged images (right). (A) NG2-expressing OPC. (B) CNPase-expressing myelinating oligodendrocyte, with axons labelled for NF 160/200. (C) Myelinating oligodendrocyte expressing MBP. (D) NF 160/200-expressing DRG neuron. (E) GFAP-expressing astrocyte. (F) SCIP-expressing satellite cell. (G–J) Specimen currents and mean currents (number of cells shown on bars) evoked at −64 mV by 60 µM NMDA and 30 µM kainate in the absence or presence of NRG, in (G–H) oligodendrocyte precursor cells and (I–J) differentiated oligodendrocytes in the process of myelinating neurons. (K–N) Mean currents in (K) DRG neurons, (L) interneurons, (M) astrocytes, and (N) satellite cells. Internal solution had E_Cl_ = 0 mV. See also Figure S4.

To assess which cells' glutamate receptors might be regulating myelination in the presence of NRG, we recorded from the cells in the cocultures at the time when myelination was measured (3 wk after adding the OPCs), using postrecording antibody labelling (Figure 4A, Figure 5A–F) and membrane I-V relations (Figure S4) to identify them. In cultures exposed to NRG, the membrane resistance of oligodendrocyte lineage cells was not altered (OPCs, control 836±369 MΩ versus NRG 894±483 MΩ, *p* = 0.92; differentiated oligodendrocytes, control 130±25 MΩ versus NRG 208±65 MΩ, *p* = 0.20). However, with added NRG the NMDA-evoked currents at −64 mV in oligodendrocyte precursor cells and in differentiated oligodendrocytes were ∼6-fold larger than in cells not exposed to added NRG (*p* = 0.02 and *p* = 0.03, respectively; Figure 5G–J). In contrast, kainate-evoked currents (mediated by AMPA/kainate receptors) were not significantly affected by NRG in either OPCs (*p* = 0.48) or differentiated oligodendrocytes (*p* = 0.73; Figure 5G–J). NRG did not significantly alter the size of NMDA- or kainate-evoked currents in DRG neurons (*p* = 0.75 and *p* = 0.93, respectively; Figure 5K), interneurons (*p* = 0.30 and *p* = 0.92; Figure 5L), astrocytes (*p* = 1 and *p* = 0.78; Figure 5M), or satellite cells (*p* = 0.62 and *p* = 0.52; Figure 5N) that were also present in the cultures. These data indicate that NRG specifically upregulates NMDA responses in oligodendrocyte lineage cells, providing a mechanism for these cells to become more sensitive to glutamate released from active axons.

**Figure 6:**
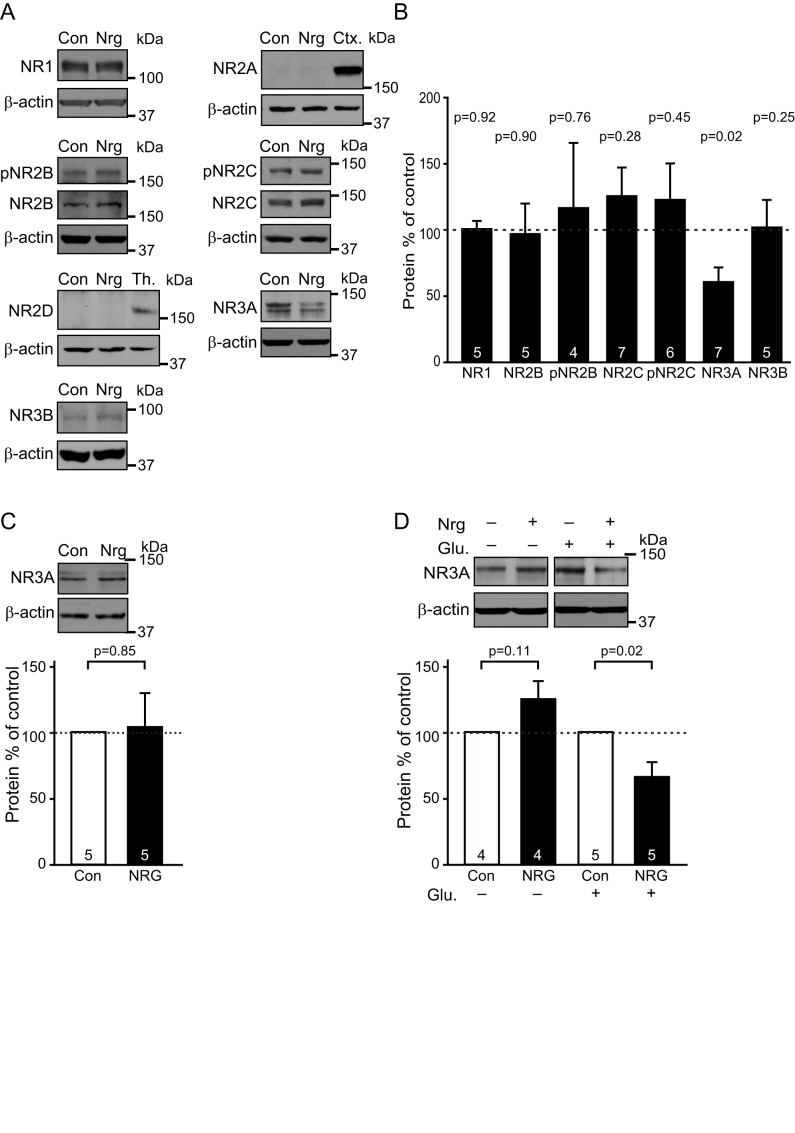
Expression of NMDA receptors. (A) Western blots of control and NRG-treated cocultures for NR1, NR2B and its phosphorylated form (pNR2B), NR2C and its phosphorylated form (pNR2C), NR3A and NR3B, as well as for NR2A and NR2D compared to their respective positive controls of rat cortex (Ctx) and thalamus (Th); β-actin acts as a loading control throughout. (B) Densitometric quantification of subunit protein levels in cocultures (normalized to β-actin) in NRG normalized to the levels in control (NR2A and NR2D levels were undetectable). (C) Western blot of control (Con) and NRG-treated pure DRG cultures for NR3A and (below) densitometric quantification of subunit protein levels (normalized to β-actin and then to control). (D) Western blot for NR1 and NR3A of control (Con) and NRG-treated (for 6 d) pure OPC cultures, treated (+) or not treated (−) with 20 min glutamate (Glu, 100 µM) stimulation every day, with densitometric quantification of subunit protein levels (normalized to β-actin and then to control). The *p* values over the bars, in (B) from Holm–Bonferroni corrected *t* tests and in (C and D) from one-sample Student *t* tests, compare with control; numbers of experiments shown on bars.

NMDA receptors in oligodendrocyte lineage cells have been suggested to mainly comprise NR1, NR2C, and NR3A subunits [18]–[20],[29]. NR2B and NR2C are the main subunits known to be phosphorylated (by Fyn and Akt, respectively) downstream of NRG and other growth factor signalling [30],[31], and NR2C phosphorylation promotes subunit trafficking to the plasma membrane [31]. We therefore investigated whether the increase in NMDA responses reflected altered receptor subunit expression or phosphorylation, focussing on the possible role of NR2B, NR2C, and NR3A subunits (in fact, no NR2A or NR2D and very little NR3B were detected in the cocultures). Western blotting revealed unchanged levels of NR1, of NR2B and its phosphorylated form, of NR2C and its phosphorylated form, and of NR3B (Figure 6A,B). However, the level of NR3A protein was down-regulated by 40% in the presence of NRG (*p* = 0.02; Figure 6A,B). NRG did not affect the NR3A protein level in pure DRG cultures (*p* = 0.85; Figure 6C), but downregulated NR3A by 33% in pure OPC cultures provided that glutamate was also added to mimic glutamate release from DRG axons (*p* = 0.02; Figure 6D and see Discussion). Removal of NR3A subunits from NMDA receptors composed of NR1, NR2, and NR3 subunits has previously been reported to increase their single channel current, their trafficking to the surface membrane, and their calcium permeability, and thus to increase NMDA-evoked currents 2.8-fold [29],[32],[33]. Consequently, a down-regulation of NR3A synthesis or an increase of its degradation could account for the increased NMDA receptor current seen in NRG.

### Only Activity-Dependent Myelination Is Integrin Dependent

**Figure 7:**
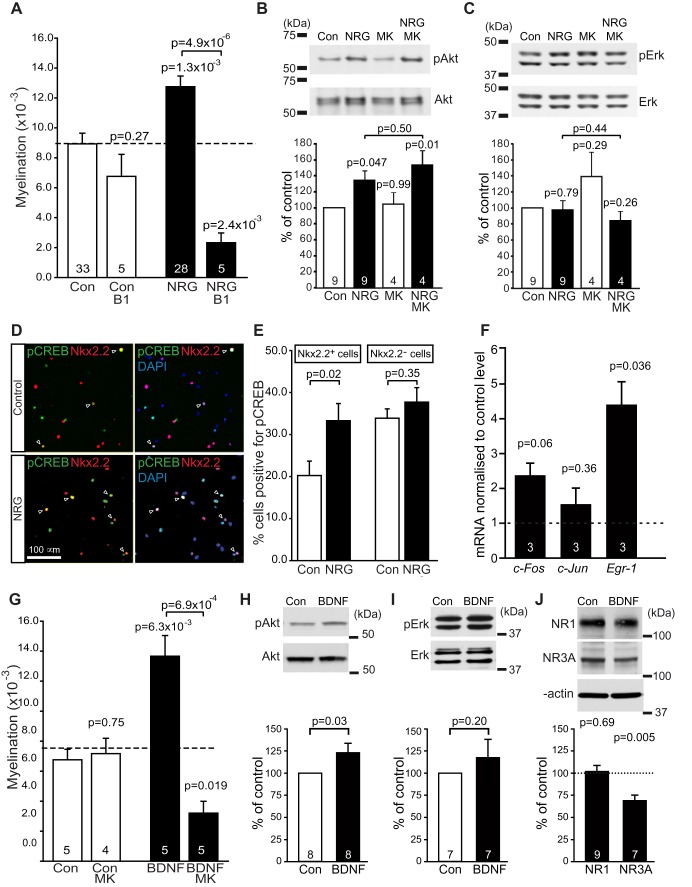
Signalling underlying the effects of NRG. (A) Effect of blocking integrin function with an antibody to the β_1_ subunit (β1) in the absence and presence of NRG (number of experiments shown on bars). ANOVA across all conditions gave *p*<0.0001. The *p* values above each bar compare with control. (B–C) Western blot of control (Con) and NRG cocultures (numbers of co-cultures shown on bars), in the absence and presence of MK-801, for phosphorylated (pAkt, pERK) and total Akt and ERK, with level of phosphorylated enzyme normalized to total enzyme in bar charts below. (D) Cocultures labelled for nuclei with DAPI, and with antibodies to Nkx2.2 (for late OPCs/immature oligodendrocytes) and pCREB (arrows show cells expressing both). (E) Percentage of cells expressing and not expressing Nkx2.2, which label for pCREB (in 29 control and 29 NRG fields of view, including 26,261 control and 21,518 NRG cells). (F) Change in immediate early gene expression mRNA level (by qPCR) in NRG-treated cocultures, normalized to control levels. (G) Effect of BDNF and MK-801 on myelination. One-way ANOVA gave *p*<0.0001 (the potentiation of myelination by BDNF here cannot be directly compared with that for NRG in Figure 2A because the experiments were not done on the same set of cocultures). (H–I) Western blot of control (Con) and BDNF cocultures, for phosphorylated (pAkt, pERK) and total Akt and ERK, with level of phosphorylated enzyme normalized to total enzyme in bar charts below. (J) Western blots of control (Con) and BDNF-treated cocultures for NR1 and NR3A, with densitometric quantification of subunit protein levels in bar graph below. The *p* values over single bars compare with control (Con). The *p* values are from Holm–Bonferroni corrected *t* tests in (A and G), Dunnett's post hoc tests in (B and C), and Student's *t* tests in (H–J). Number of cultures are shown on bars.

Myelination depends on integrins [34], and integrins modulate NMDA receptor expression [35]. We therefore tested whether the NRG-evoked switch from activity-independent to NMDA receptor-dependent myelination depends on integrins, using an antibody to β_1_ integrin (1 µg/ml) to block its function [36]. This had no effect on myelination without added NRG (*p* = 0.27), but in the presence of NRG it reduced myelination by 82% (*p* = 5×10^−6^; Figure 7A) to only 26% of the level seen without NRG (significantly lower, *p* = 0.0024). Thus, unlike the activity-independent mode of myelination, the activity and NMDA receptor-dependent myelination induced by NRG is dependent on integrin function.

### Signalling Underlying the Effects of NRG

NRG can activate the PI3K-Akt or MAPK/Erk pathways in oligodendrocytes [37]. To test which pathway mediated NRG's effects, we measured the levels of phosphorylated Akt and Erk in the cocultures. NRG increased Akt phosphorylation by 43% (*p* = 0.047; Figure 7B), with no effect on Erk phosphorylation (*p* = 0.79; Figure 7C). In contrast in pure DRG cultures neither pAkt nor pErk levels were affected (Figure S5B,C). To test whether activation of Akt was downstream of the increased NMDA receptor activation produced by NRG, we measured the levels of pAkt and pErk in cocultures treated with MK-801 (Figure 7B,C). MK-801 had no effect on the actions of NRG on Akt: NRG added with MK-801 increased pAkt (*p* = 0.01) with no effect on pErk (*p* = 0.26), and there was no significant difference between the levels of pAkt in NRG, with or without MK-801 (*p* = 0.50). Thus, activation of Akt does not occur downstream of NMDA receptor activation (it may be upstream of the NRG-evoked increase in NMDA receptor current, or on an independent NRG-activated pathway). NRG also increased by 65% (*p* = 0.02) the proportion of Nkx2.2-expressing cells (late OPCs/immature oligodendrocytes) that expressed phosphorylated CREB (pCREB), an Akt target [38], without affecting the percentage of cells lacking Nkx2.2 that expressed pCREB (*p* = 0.35; Figure 7D,E).

Next we looked into CREB target genes. NRG increased more than 4-fold the message level for the early growth response gene *Egr-1* (*p* = 0.036; Figure 7F), a CREB target gene [39] that has been shown to be regulated by integrin β1-dependent PI3K/Akt activation [40], neuronal activity [41], activation of NMDA receptors [42], and activation of OPC glutamate receptors [43]. EGR-1 is an upstream regulator of MRF [44], an essential transcription factor needed for myelination [45]. NRG more than doubled the level of the CREB target *c-Fos*, but this did not reach significance (*p* = 0.06) and had no effect on the level of *c-Jun* (*p* = 0.36; Figure 7F).

These data suggest that NRG promotes myelination via Akt, integrin β1, NMDA receptors, CREB, and EGR-1 signalling. This is consistent with constitutively active Akt promoting myelination [46], with β1 integrin activating Akt to promote myelination [34], and with CREB activating transcription of myelin genes [39],[47].

### BDNF Also Induces NMDAR-Dependent Myelination

The role of NRG/ErbB signalling in CNS myelination is controversial, as decreasing NRG or ErbB function reduced myelination [5]–[7], while knocking NRG or ErbB out had no effect on myelination [9]. Because of the importance of myelination, there may be redundancy in the mechanisms regulating it. Like NRG, BDNF increases NMDA receptor currents in neurons by upregulating NR2C subunits [48], and via TrkB it can promote oligodendrocyte differentiation, expression of MBP [49], and CNS myelination [50]. We therefore tested the effect of adding BDNF (10 ng/ml, 0.7 nM) to the cocultures. BDNF increased myelination (by 102%, *p* = 0.006; Figure 7G) and MBP expression (*p* = 0.048; Figure S1G) and, as for NRG, made myelination become dependent on activation of NMDA receptors (Figure 7G). With BDNF present, MK-801 reduced myelination by 76% (*p* = 6.9×10^−4^), to only 47% of its value without BDNF (*p* = 0.019). Like NRG, BDNF induced phosphorylation of Akt but not Erk (Figure 7H–I; *p* = 0.03 and *p* = 0.2, respectively), and downregulated expression of the NR3A subunit but not the NR1 subunit of NMDA receptors (Figure 7J; *p* = 0.005 and *p* = 0.69, respectively). Thus, by acting through similar signalling pathways, BDNF can also switch oligodendrocyte myelination to a pathway that depends on glutamate release activating NMDA receptors. Consequently, when the NRG/ErbB pathway is knocked out, myelination may still occur [9] not only via the activity-independent mode of myelination (Figure 2A) but also because of compensatory BDNF signalling (see Discussion). 

### Remyelination *in Vivo* Is NMDA Receptor Dependent

Our demonstration of NRG- and NMDAR-dependent myelination of axons by oligodendrocytes raises the question of whether this same mechanism operates during remyelination after white matter damage. A lack of NRG signalling has been suggested to result in poor remyelination in multiple sclerosis [51],[52]. The data above suggest this may reflect a lack of NRG-dependent upregulation of NMDA receptors in oligodendrocyte lineage cells, but NMDA receptor deletion or block has been variously reported to either have no effect on loss of myelin in the experimental autoimmune encephalomyelitis model of multiple sclerosis [24] or to delay remyelination after cuprizone demyelination [25]. We therefore tested whether successful remyelination is dependent on NMDA receptor activation, in OPCs that are recruited to remyelinate axons in a focal toxin-induced demyelinated lesion in the rat caudal cerebellar peduncle. This demyelination model provides successful spontaneous remyelination that occurs with a clear temporal separation from the acute demyelination [53].

**Figure 8:**
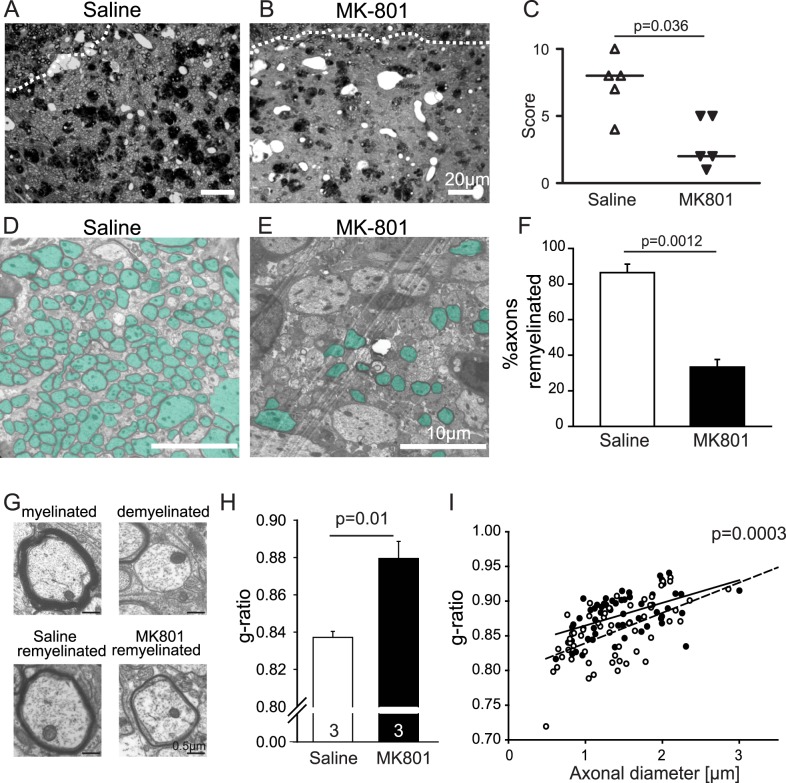
Remyelination is dependent on NMDA receptor activation. (A–B) Semithin sections of lesioned caudal cerebellar peduncle (CCP), 21 d postlesion, infused with saline (A) or MK-801 (50 µM; B) for 18 d. The dotted white lines mark the lesion edge. (C) Ranking of remyelination; each symbol represents one animal. Higher ranks represent more remyelination. The *p* value from Mann–Whitney U test. (D, E) Higher magnification picture shows that fewer axons are remyelinated in lesions treated with MK-801 (remyelinated axons are coloured green). (F) Mean percentage of axons remyelinated averaged over 20 areas in each of three lesions for each condition. (G) Specimen images of a normal myelinated axon, a demyelinated axon, and remyelinated axons in lesions infused with saline or MK-801. (H–I) Mean g-ratio of all axons, and mean g-ratio at all diameters, are higher with MK-801 present (student *t* test, *n* = 3).

An intracerebral implanted cannula, connected to an osmotic minipump, infused into the lesion either 0.9% saline or 50 µM MK-801 (at a flow rate of 0.11 µl/h) from the 3rd day postlesion (the timepoint when OPCs enter the lesion, [54]) until the animal was sacrificed at 21 d postlesion. Analysis of semithin sections stained with toluidine blue showed that there was no difference in lesion size (saline, 0.28±0.04 mm^2^; MK-801, 0.22±0.04 mm^2^, *p* = 0.28) nor axon density (saline, 50,800±2,100 axons/mm^2^; MK-801, 47,500±1,900 axons/mm^2^, *p* = 0.27) between the conditions, but a blinded analysis ranking remyelination revealed that blocking NMDA receptors with MK-801 significantly inhibited remyelination (*p* = 0.036; Figure 8A–C). Moreover, at the ultrastructural level, it was clear that fewer axons were remyelinated when NMDA receptors were blocked (*p* = 0.0012; Figure 8D–F), and the g-ratio (the ratio of axon diameter to outside diameter of the myelin) of remyelinated axons was higher in MK-801– compared to saline-infused lesions (*p* = 0.01; Figure 8G–I), showing that the myelin was thinner. Thus, efficient remyelination depends on activation of NMDA receptors.

## Discussion

The speeding of action potential propagation produced by myelination of axons is crucial for CNS function. We have shown that, in the absence of added NRG, myelination takes place by a mechanism that is independent of neuronal activity and glutamate release, because it is unaffected by blocking action potentials or ionotropic glutamate receptors. With NRG added, myelination is accelerated and increased, this effect is dependent on action potentials, and blocking NMDA receptors greatly reduces myelination (to well below the level seen in the absence of added NRG; Figures 1 and 2). Thus, the key result of this article is that NRG does not just speed and increase myelination; it produces a switch in the mechanism of myelination, from a default programme that is independent of neuronal activity (which allows oligodendrocytes to ensheath even fixed axons [12]) to a programme that depends on activation of NMDA receptors in oligodendrocyte lineage cells, presumably by glutamate released from active axons [14]–[18],[55] (although we cannot rule out a contribution of glutamate release, either tonic or activity-induced, from other cells). This switch implies a suppression of the default programme when the activity-dependent programme is activated by NRG. Furthermore, the influence of NMDA receptors on myelination is not limited to myelination occurring during normal development, – because remyelination of demyelinated axons in the cerebellar peduncle *in vivo* also depends on NMDA receptor activation (Figure 8).

Adding NRG increases 6-fold the NMDA-evoked currents in oligodendrocyte precursor cells and in differentiated oligodendrocytes, but does not significantly alter NMDA-evoked currents in DRG neurons, nor NMDA- or kainate-evoked currents in any other cell type in the culture (Figure 5), nor the action potential firing of DRG axons (Figure 4). We therefore attribute the NRG-induced activity dependence of myelination to a potentiation of NMDA receptor signalling in oligodendrocyte lineage cells, making the cells more sensitive to axonal activity. This potentiation may largely reflect a decreased protein level of NR3A subunits (Figure 6). However, the 6-fold increase in NMDA receptor-mediated current that we find is larger than the 2.8-fold increase reported in neurons when NR3A is knocked out [32]. Consequently, because of possible obscuring of changes in oligodendrocyte protein levels by neuronal NMDA receptor protein in the cocultures, we cannot rule out a contribution to the increase in NMDA-evoked current from alterations of the levels of other NMDA receptor subunits in oligodendrocyte lineage cells, of which upregulation of NR2C [21] and/or NR2B [30] subunits are the most likely candidates.

In the presence of NRG, blocking action potentials reduces myelination less than does blocking NMDA receptors (Figure 2A), despite the fact that it is presumably action potentials that release the glutamate that activates the NMDA receptors. Thus, in NRG, TTX reduces myelination to a value close to that occurring without NRG present (Figures 2A and S5A). This may reflect action potential evoked glutamate release being needed for NRG to increase NMDA receptor currents, so that the switch to NMDA receptor-dependent myelination does not occur in the absence of action potentials. This is likely since NMDA receptor activation is needed both for removal of NR3A subunits from the surface membrane [56] and for NRG to upregulate NR2C subunits in neurons [21]. Consistent with this, blocking the glutamate site on NMDA receptors with D-AP5 reduced myelination in NRG less than did MK-801 or 7-chlorokynurenate, which block at other sites on NMDARs (Figure 2A), and in pure OPC cultures, NRG downregulated NR3A only if glutamate was also added (Figure 6D). The inhibitory effect of NBQX in NRG in Figure 2A may similarly reflect AMPA/kainate receptor activity maintaining neuronal firing, which evokes the glutamate release needed to upregulate NMDA currents [21],[56], or could reflect AMPA/kainate receptor-mediated depolarisation increasing current flow through NMDA receptors [18].

The exact sequence of signalling steps in oligodendrocytes that upregulates NMDA receptor currents, and thus speeds, increases, and confers activity-dependence upon myelination, remains to be defined, although we have shown that Akt, integrin β_1_, CREB, and EGR-1 are involved (Figure 7). Since NRG activates Akt (Figure 7B) and Fyn [30] and these kinases are known to alter NMDA receptor subunit expression and trafficking [30],[31] and to promote myelination [16],[46],[57], it is an attractive idea that NRG may initially activate Akt and/or Fyn, and that this action is potentiated by integrins (Figure 7A) [34],[35],[57].

Our results help resolve three controversies in the field. The first concerns the role of NRG in CNS myelination. Decreasing NRG or ErbB function reduces myelination [5]–[7], and in the prefrontal cortex, NRG's control of myelination is regulated by social interactions [58]. However, knocking NRG or ErbB out had no effect on myelination, even though overexpressing NRG increased myelination [9]. Our data predict that myelination can appear to be unaffected when NRG-ErbB signalling is abolished, for three reasons. First, the activity-independent mode of myelination (Figure 2A) should still occur. Second, when myelination levels are high, as may be the case *in vivo*, NRG only modestly increases myelination (Figure 2D), yet it still makes myelination become very highly dependent on neuronal activity releasing glutamate to activate NMDA receptors (Figure 2E). Third, our data indicate redundancy in the growth factors that switch oligodendrocytes between the two myelination modes. Both NRG and BDNF alter NMDA receptor expression in oligodendrocyte lineage cells, induce Akt activation but not Erk activation (although promotion of myelination by BDNF can also involve Erk activation [59]), and promote myelination, and both switch oligodendrocytes to the NMDA receptor-dependent mode of myelination (Figures 2A and 7G). Since not only ErbB receptors for NRG but also TrkB receptors for BDNF (including full length receptors [49],[50],[60]–[62]) can be expressed by oligodendrocytes, our data suggest that the failure of NRG or ErbB knockout to affect CNS myelination may, in part, reflect another growth factor (BDNF) and its receptor (TrkB) acting to replace the NRG/ErbB system.

The second controversy concerns the role of NMDA receptors in CNS myelination. Deleting the NMDA receptor NR1 subunit from oligodendrocyte lineage cells has been reported to have no effect on myelination [23],[24], yet activation of NMDA receptors by glutamate released from active axons is reported to promote MBP translation and myelination [16]. Interestingly, in another oligodendrocyte-specific NR1 knockout, deletion of NMDA receptors slowed myelination in the optic nerve [63], consistent with the acceleration of myelination that we observe when NMDA receptor currents are increased in the presence of NRG (Figure 2C). This effect of NMDA receptors was proposed to be caused by NMDA receptor activation upregulating glucose transporters in oligodendrocyte lineage cells (as was previously shown to occur in an Akt-dependent manner in neurons; [64]) to provide energy for myelination [63].

The third controversy concerns whether myelination is activity dependent or independent. Rearing rodents in the dark from birth, or injecting TTX into the optic nerve, can reduce or alternatively have no effect on myelination [10],[65]–[67]. Similarly, electrical activity [10],[16], or application of factors it releases [11], can promote myelination, and blocking neuronal activity with TTX in myelinating cortical cultures reduced myelination [10], whereas in spinal cord cultures TTX did not affect myelination [68]. Presumably, these differences may reflect the existence of the two alternative myelination programmes that we have characterised (whereby in the absence of added NRG, TTX has no effect on myelination, but in NRG-treated cultures, it reduces myelination by 50%; Figure 2A). Whether myelination is predominantly activity dependent or independent may depend on the amount of NRG or BDNF (or other molecules [69],[70]) being expressed or released [21],[71]. In this context it is worth noting that, although we have discussed our results as reflecting the presence or absence of NRG, in the absence of added NRG, there will be some level of endogenous NRG release, and it may be better to think of whether the NRG level is low or high as determining the main myelination mode that occurs. It is also possible that the switch from one mode to the other occurs gradually as the NRG level rises.

The existence of two alternative myelination programmes regulated by NRG level, independent of, and depending on, action potentials and NMDA receptors, may reflect the evolutionary importance, but also the metabolic cost, of myelination. One can speculate that early in development it may be important to myelinate whatever axons are present, irrespective of their impulse traffic. However, once a significant density of axons is present, because myelination involves considerable investment by the oligodendrocyte in lipid production [72], it is more efficient for myelination to be focussed on axons that have a high impulse traffic, rather than on inactive axons. The NRG- and NMDA receptor-dependent mode of myelination may dominate later in development, since late myelination in the prefrontal cortex depends on NRG signalling [58] and adult remyelination depends on NMDA receptor activation (Figure 8).

NRG is a susceptibility gene for schizophrenia [22],[73], and NMDA receptors are also implicated in this disease [74]. NRG affects NMDA receptor expression in the grey matter [21],[22], where the defect underlying schizophrenia is usually assumed to occur. However, correct myelination is essential for normal cognitive function, and the interaction of NRG and NMDA receptors to control myelination allows us to speculate that there could perhaps be a white matter explanation for the linkage of NRG and NMDA receptors to schizophrenia. Furthermore, NRG expression is reduced in multiple sclerosis lesions [52], and adding NRG has been suggested to promote remyelination in a mouse model of multiple sclerosis [51], suggesting that NRG- and NMDA receptor-dependent remyelination may be important after pathology. Consistent with this we have shown that, *in vivo* in the cerebellar peduncle, successful remyelination is reduced when NMDA receptors are blocked (Figure 8) and similar results have been found for remyelination in the corpus callosum [25]. Promoting NRG- and NMDA receptor-dependent myelination may, therefore, be a useful therapeutic strategy for increasing CNS remyelination in disease.

## Material and Methods

### DRG-Oligodendrocyte Cocultures

These were made as described previously [8]. Briefly, DRG cells from E14–E16 rats were cultured for 2 wk, and then cultured oligodendrocyte precursor cells (OPCs) from P0–P2 rats were plated on top of them [8]. One coverslip was analysed for each drug condition per dissection. Three weeks later, the cocultures were fixed, labelled, and images were taken of each coverslip to assess myelination (see detailed description below) [8]. NRG (Lab Vision) or BDNF (R&D systems) and receptor blockers (Tocris) were applied with the OPCs (except when TTX was added 3 d later than the NRG and OPCs) and were included in medium changes twice per week thereafter. Culture medium contained 0.8 mM MgCl_2_ and 0.4 mM glycine, but no added glutamate.

### DRG Cultures

As described above, DRG cells from E14–E16 rats were cultured for 2 wk, then put in myelination medium (as for cocultures but lacking OPCs), for 3 wk, with medium changes twice per week thereafter.

### Oligodendrocyte Cultures

These were as described previously [8]. Briefly, purified oligodendrocyte precursors were obtained with minor modifications [36] of the method of McCarthy and de Vellis [75]. They were cultured in myelinating medium (the same as for the cocultures) for 6 d, either with or without 20 min 100 µM glutamate stimulation every day for the last 5 d *in vitro*.

### Quantification of Myelination

For each coverslip, 30 randomly located images were taken of MBP and NF staining (using either a 10× objective, with a field of view of 843 µm×636 µm, or a 20× objective, with a field of view of 709 µm×530 µm). Within each image, myelination was quantified by counting the number of myelinating MBP-positive oligodendrocytes (Figure 1B,C) as a percentage of the total number of MBP-positive oligodendrocytes [8]. The density of axons was expressed as the percentage of the same area occupied by NF (calculated from a binarised NF image using ImageJ software, version 1.34s). There was no correlation between oligodendrocyte density and axon density. The fraction of myelinating cells was plotted against the axon density for the different images, and fitted assuming a linear dependence [8] of *fraction of oligodendrocytes myelinating* (*F*) on *axon density* (*D*),
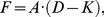
(1)where *K* is a free constant and the slope *A* is the measure of myelination plotted in Figure 2, Figure S2A, Figure S3A, and Figure 7. Fitting data in control and NRG conditions gave values for *K* that were not significantly different (12.9±1.3%, *n* = 32 experiments, for control, and 12.6±1.8%, *n* = 27, for NRG; *p* = 0.88). This quantification of myelination gave results similar to those obtained using other assumed dependencies of *fraction of oligodendrocytes myelinating* (*F*) on *axon density* (*D*) as follows:

1. A linear dependence above a threshold density: 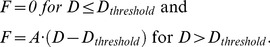
(2)Fitting data in control and NRG conditions with *D_threshold_*, a free parameter gave thresholds that were not significantly different from *D_threshold_* = 17% (17.9±1.8%, *n* = 32, *p* = 0.62 for control, and 17.2±2.4%, *n* = 27, *p* = 0.93 for NRG), so *D_threshold_* was set to 17%; we refitted the data, and used the value of *A* as a measure of myelination (Figure S2B, Figure S3B).
2. A power law:
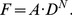
(3)Fitting data in control conditions or in the presence of NRG with *N* a free parameter gave best fit powers not significantly different from *N* = 2 (2.08±0.14, *n* = 32, *p* = 0.57, and 2.10±0.16, *n* = 27, *p* = 0.54, respectively), so we fixed the power at 2, re-fitted the data, and then used the amplitude (*A*) of the best fit curve as the measure of myelination (Figure S2C and Figure S3C).

Irrespective of the quantification used, the bar charts in different conditions (Figure S3A–C) gave the same results: blocking action potentials or NMDA receptors in control conditions had no effect on myelination, NRG significantly increased myelination, and in the presence of NRG blocking action potentials produced a significant reduction of myelination, while blocking NMDA receptors reduced myelination so strongly that it was significantly reduced below the control level with no NRG. If, instead of fitting the dependence of myelination on local axon density, we simply counted the fraction of oligodendrocytes that myelinated, by averaging over all the sampled images, then the results were broadly similar, except that NMDA receptor block in control conditions produced a reduction of myelination of borderline significance (Figure S3D).

### Electrophysiology

Cells were whole-cell patch-clamped [14],[18] at room temperature (21–24°C) in cocultures 3 wk after the OPCs were added. Electrodes contained solution comprising (mM) either 126 CsCl, 4 NaCl, 10 HEPES, 5 EGTA, 4 MgATP, 0.5 Na_2_GTP, 12 phosphocreatine, 2 K-Lucifer yellow, pH set to 7.3 with CsOH (E_Cl_ = 0 mV), or 130 Cs-gluconate, 4 NaCl, 0.5 CaCl_2_, 10 HEPES, 10 BAPTA, 4 MgATP, 0.5 Na_2_GTP, 2 K-Lucifer yellow, pH set to 7.3 with CsOH (E_Cl_ = −88 mV). Series resistance was 5–20 MΩ, and electrode junction potentials were compensated. Cultures were superfused at 24±1°C with HEPES-buffered solution containing (mM) 144 NaCl, 2.5 KCl, 10 HEPES, 1 NaH_2_PO_4_, 2.5 CaCl_2_, 10 glucose, 0.1 glycine (to coactivate NMDA receptors), 0.005 strychnine (to block glycine receptors), pH set to 7.4 with NaOH, bubbled with 100% O_2_. Cells were identified (Figures 4A and 5A–F) by their postrecording dye-fill morphology [14],[18], confirmed by antibody labelling against the proteoglycan NG2 to identify oligodendrocyte precursors (17/17 tested labelled for NG2), against CNPase (11/11), MBP (10/10), MOG (4/4), or GalC (11/11) for differentiated oligodendrocytes, against GAD 65/67 for interneurons (21/21), against NF 160/200 (13/13) for DRG neurons, against GFAP for astrocytes (11/11), and against SCIP for satellite cells (10/10). In addition we checked that each recorded cell had the electrophysiological properties expected for its class: specimen responses to voltage steps from the resting potential are shown for each cell class in Figure S4. OPC morphology cells, including those confirmed as being OPCs by virtue of their labelling with antibody against NG2, fell into two classes [14] with (57% of cells) and without (43% of cells) voltage-gated Na^+^ current (Figure S4A–D), and had a steady-state input resistance of ∼800 MΩ. Mature myelinating oligodendrocytes had a roughly ohmic and time-independent I-V relation in the physiological range (Figure S4E), with a much lower input resistance of ∼160 MΩ. Interneurons and DRG neurons both showed a voltage-gated Na^+^ current on depolarization, which was too large to clamp well, so the I-V relations showed current oscillations reflecting uncontrolled action potentials occurring (Figure S4F,G). Both satellite cells and astrocytes showed roughly ohmic and time-independent I-V relations in the physiological range (Figure S4H,I) with a mean input resistance near the resting potential of ∼900 and ∼15 MΩ, respectively. 

### Synaptic Current Analysis

A synaptic current was defined to occur if its amplitude was >3 times the standard deviation of the baseline current noise and its 10%–90% decay time was longer than its rise time. Events were detected and analysed with pClamp 10 (Axon Instruments).

### Immunohistochemistry

Cultures were fixed at 21°C for 20 min in 4% PFA and incubated for 1 h in 0.1% Triton X-100, 10% goat serum in phosphate-buffered saline at 21°C, then with primary antibody at 21°C for 2 h or for overnight at 4°C, and then for 1 h at 21°C with secondary antibody. Primary antibodies were guinea pig NG2 (a kind gift from W.B. Stallcup, 1∶100), rabbit NG2 (Chemicon, 1∶300), rabbit Olig2 (a kind gift from D. Rowitch, C.D. Stiles & J. Alberta, 1∶20,000, or Chemicon, 1∶1,000), rabbit Caspr (a kind gift from D. Colman & J. Huang, 1∶500), rabbit SCIP (a kind gift from J.R. Bermingham, 1∶100), rabbit GalC (Sigma, 1∶100), mouse NF 160/200 (Sigma, 1∶1,000), rat MBP (Serotec, 1∶100), mouse MOG (Sigma, 1∶100), mouse CNPase (Sigma, 1∶100), rabbit GAD 65/67 (Chemicon, 1∶100), rabbit GFAP (Dako, 1∶500), chicken P0 (Aves Labs, 1∶500), mouse Nkx2.2 (DSHB, 1∶120), CD11b (Serotec, 1∶50), and rabbit pCREB (Cell Signalling, 1∶50). Alexa 488–conjugated isolectin B4 (Invitrogen, 1∶100) was used to label microglia. DAPI (Sigma, 20 µM) was used to label nuclei. Secondary antibodies (goat) were for rabbit (Molecular Probes, 1∶1,000), rat IgG (Molecular Probes, 1∶1,000), mouse IgG (Molecular Probes, 1∶1,000), chicken (Jackson Lab, 1∶1,000), and guinea pig (Jackson Lab, 1∶100).

### Western Blots

For protein analysis, cultures were scraped from 22 mm coverslips, 6 well plates, or 10 cm dishes and lysed mechanically in solution containing 0.1 M phosphate buffered saline (PBS), 10% or 20% (w/v) sucrose and Halt protease, and phosphatase inhibitor cocktail (Thermo Scientific). Protein content was determined by Bradford assay and quantified using a standard curve obtained from Quick Start BSA protein standards (Bio-Rad). Equal amounts of protein (8 or 10 µg) from samples were resolved on 4–%12% NuPage Novex Bis-Tris mini gels (Invitrogen), with prestained molecular weight protein standards (Bio-Rad). Proteins were transferred to a nitrocellulose membrane (0.45 µm, GE Healthcare) using a wet transfer system. Nitrocellulose membranes were blocked for 1 h at room temperature with 3% BSA in PBS with 0.1% Tween-20 (PBS-T). Immunoblots were then incubated overnight at 4°C with goat anti-Akt (Santa Cruz, 1∶1,000), rabbit anti-phosphorylated-Akt^Ser473^ (Cell Signalling, 1∶1,000), rabbit anti-phosphorylated-ERK1/2^Thr202/Tyr214^ (Cell Signalling, 1∶1,000), mouse anti-ERK1/2 (Cell Signalling, 1∶2,000), rabbit anti-MBP (Sigma, 1∶3,000), mouse anti-NR1 (Millipore, 1∶1,000), rabbit anti-NR2A (Millipore, 1∶1,000), rabbit anti-NR2B (Abcam; 1∶1,000), rabbit anti-phosphorylated NR2B^Tyr1472^ (Millipore, 1∶1,000), rabbit anti-NR2C (Millipore, 1∶1,000), rabbit anti-phosphorylated NR2C^S1096^ (a kind gift from Katherine W. Roche, NINDS), rabbit anti-NR3A (Millipore, 1∶500), rabbit anti-NR3B (Millipore, 1∶300), rabbit anti-NR2D (Abcam, 1∶300), or mouse anti-β-actin (Sigma, 1∶100,000) in 3% BSA in PBS-T. This was followed by incubation with the secondary horseradish peroxidise-linked anti-rabbit, anti-mouse, or anti-goat antibodies (1∶1,000, Dako) for 1 h at room temperature in 3% BSA in PBS-T. Immunoreactive proteins were visualised with enhanced chemiluminescence (GE Healthcare). When phosphorylated and total amounts of the same protein were measured, this was done by first probing with the antibody to the phosphorylated protein, then stripping the membranes to remove the antibody, and reprobing with antibody recognizing both phosphorylated and unphosphorylated forms. Stripping membranes was performed with 50 mM dithiothreitol, 50 mM Tris (pH 6.8), and 2% SDS at 70°C for 30 min and washing three times in PBS-T before blocking and incubating with the primary antibody. Densitometric analysis was conducted using ImageJ gel analysis software (version 1.43u). For each sample, pAkt, pERK, pNR2B, and pNR2C signals were normalised to the total levels of their respective proteins present. The levels of nonphosphorylated proteins were normalized against β-actin.

### Quantitative RT-PCRs

RNA was extracted from DRG-OPC cocultures 21 d after addition of OPCs using an RNeasy mini kit (QIAGEN). RNA was converted to cDNA using a SuperScript First-Strand Synthesis System for RT-PCR (Invitrogen). qPCR was performed with a QuantiFast SYBR Green PCR Kit (QIAGEN) using a LightCycler 480 II. Samples were normalised to GAPDH levels using commercially available QuantiTect Primers (QIAGEN) for analysis.

### Induction of Focal Demyelination

Female Sprague-Dawley rats aged 9–12 wk of age (200–225 g) were used for remyelination studies, which were performed in compliance with UK Home Office regulations. Focal demyelination was induced unilaterally by stereotaxically injecting 4.0 µl of 0.01% ethidium bromide (w/v) in saline into the caudal cerebellar peduncle (CCP) (as described previously [53]). For continuous local delivery of MK-801 (50 µM, Tocris, in 0.9% saline) or 0.9% saline (Vetivex) into the demyelinated lesion, an osmotic minipump with a reservoir volume of 100 µl and a flow rate of 0.11 µl/h (Alzet Micro-Osmotic Pumps, model 1004, DURECT Corporation) was attached through a vinyl tube spacer (Plastics One Inc., Roanoke, Virginia) to a 30 gauge (6.5 mm) cannula implanted just above the lesion. The length of the tube was cut to 2.3 cm, to ensure that drug delivery into the lesion did not occur until the 3rd day postlesion (the start of the OPC recruitment stage [54]). The minipump was placed subcutaneously, and the cannula was fixed to the skull with cyanoacrylate gel adhesive (applied under the base of the cannula head before insertion), as well as two anchoring screws and dental acrylic cement (a 1∶1 volume mix of Paladur powder and liquid; Heraeus Kulzer). Rats were randomly assigned to treatment (50 µM MK-801) or control groups (0.9% saline infusion). Animals were sacrificed 3 wk after lesion induction.

### Electron Microscopy

Co-cultures were fixed at room temperature for 1 h in PBS containing 2.5% glutaraldehyde (Agar Scientific) and 0.72 mM CaCl_2_. The cocultures were scraped off the coverslips, placed into 1.5 ml tubes with 250 µl of PBS, and centrifuged at 800 *g* for 3 min. For lesion samples, animals were perfusion fixed with 4% glutaraldehyde (Agar Scientific) in phosphate buffer containing 0.72 mM CaCl_2_. All samples were then left to fix in 2% osmium tetroxide (Oxkem Ltd) in phosphate buffer at 4°C overnight. This was followed by dehydration in 70% ethanol for 15 min, 95% ethanol for 15 min, and 100% ethanol for 3×10 min, then the tissue was placed in propylene oxide for 2×15 min, and then left for at least 3 h in a 50%/50% mixture of propylene oxide and resin mix (containing by volume: 49% TAAB embedding resin, 33% DDSA, 16% MNA, 2% DMP-30; TAAB Laboratories Equipment Ltd.). They were then transferred to 100% resin mix overnight. New resin was made and the samples left in it for at least 6 h before being placed in embedding capsules and further incubated at 60°C for 15–24 h until the resin was solid. Embedded samples were cut in 90 nm sections on an ultramicrotome (Reichert Ultracut E) with a diamond knife (Diatome) and visualised using a Transmission Electron Microscope (Hitachi H600). Images were developed on electron microscope film 4489 (Kodak), then scanned at high resolution (12,800 dpi×12,800 dpi), and analyzed with ImageJ software, version 1.34s.

### Histological Analysis of Demyelination and Remyelination

Animals were perfused with 4% glutaraldehyde (in phosphate buffer with 0.72 mM CaCl_2_), and the brains were immersion fixed in 4% glutaraldehyde for 7 d. Tissue blocks encompassing the caudal cerebellar peduncle were cut as detailed previously [53]. While maintaining their correct orientation and sequence, blocks were further fixed in 2% osmium tetroxide (Oxkem Ltd.), dehydrated in increasing concentration of ethanol, and embedded in resin (TAAB Laboratories). One micron sections were cut and stained with toluidine blue. In these sections, remyelinated axons can be easily distinguished from normally myelinated axons outside the lesion by the thinness of the myelin sheath. Within the lesion, remyelinated axons can be distinguished from demyelinated axons because the former possess myelin sheaths recognizable as a dark staining rim around the axon.

### Statistics

Data are mean ± s.e.m. The *p* values are from ANOVA and post hoc Student's two-tailed *t* tests. For multiple comparisons *p* values are corrected using a procedure equivalent to the Holm–Bonferroni method (for *N* comparisons, the most significant *p* value is multiplied by *N*, the 2nd most significant by *N*–1, the 3rd most significant by *N*–2, etc.; corrected *p* values are significant if they are less than 0.05) or Dunnett's post hoc test. Normality of data was assessed using Shapiro–Wilk tests, and nonparametric Kruskal–Wallis and Mann–Whitney tests, which do not assume data follow a normal distribution, gave the same conclusions for significant and nonsignificant differences in all cases. For Figure 2, when the ANOVA gave an insignificant *p* value (>0.05), we used the more conservative Student's two-tailed *t* test without correction for multiple comparisons to give a lower limit to the *p* value for post hoc comparisons (i.e., the differences among the control myelination data in Figure 2A are even less significant than is indicated by the *p* values).

## Supporting Information

Figure S1 **Characterization of myelination in the co-cultures.** (A) Electron microscopic image of myelination in the cocultures showing formation of compact myelin. (B) Labelling of coculture for Caspr shows formation of axon-oligodendrocyte junctions at the end of internodes. Note that some axons have single internodes so that only one Caspr-labelled region is visible at a heminode (open arrows), while some have multiple adjacent internodal segments, so that two Caspr-labelled regions are visible at nodes (filled arrows). (C) Enlarged view of a node of Ranvier labelled for Caspr. (D) DRG cells with no added OPCs show no myelination, ruling out the possibility of myelination by Schwann cells or precursors added with the DRG cells. Blue is DAPI to label nuclei, red is NF 160/200, and green is MBP (which is absent). (E) Myelination is by MBP-expressing OPCs and not P0-expressing Schwann cells. Mauve is NF, green is MBP, and red is P0 protein (a component of myelin made only by Schwann cells, which is absent). (F) The processes of DRG cells but not of interneurons become myelinated. Red is NF, green is MBP, and white is the GABA synthesizing enzyme glutamate decarboxylase (GAD 65/67). (G) Western blots of control (Con), NRG-treated and BDNF-treated cocultures for MBP (β-actin acts as a loading control), and densitometric quantification of subunit protein levels (normalized to β-actin and then to control). (H) Change in myelin gene mRNA level (by qPCR) in NRG-treated cocultures normalized to control levels. All *p* values are from one-sample Student's *t* tests. Numbers of cultures are shown on bars.
(EPS)

Figure S2 **Quantification of myelination.** Each set of graphs shows the same data (from Figure 1C–F of the main text) in control conditions, control+MK-801, NRG and NRG+MK-801, best fit with an assumed dependence of myelination (fraction of oligodendrocytes myelinating, F) on axon density (D), together with the myelination parameter (A) derived from the fit for the four conditions for this particular set of coverslips. The dependencies assumed were (A) the linear relation: F = A•(D–K), where K is a free constant, as used previously [8]; (B) the 17% threshold-linear relation: F = 0 for D≤17% and F = A•(D–17%) for D>17%; (C) the power 2 relation: F = A•D^2^.
(EPS)

Figure S3 **Myelination in various conditions quantified in different ways.** Graphs show the myelination parameter for the data analysed for Figure 2A of the main text derived from different assumed dependencies of myelination on axon density. (A) Linear relation with intercept not fixed. For no NRG, ANOVA showed no significant differences across bars (*p* = 0.72); for with NRG, ANOVA showed significant differences across bars (*p*<0.0001). (B) Threshold-linear relation with threshold of 17%. ANOVA gave *p* = 0.77 for no NRG and *p*<0.0001 for with NRG. (C) Power 2 relation. ANOVA gave *p* = 0.76 for no NRG and *p*<0.0001 for with NRG. (D) Fraction of oligodendrocytes myelinating, averaged across all images. ANOVA gave *p* = 0.096 for no NRG and *p*<0.0001 for with NRG. For all panels the *p* values, comparing data in no NRG with control, and comparing data in with NRG with NRG alone, were from Holm–Bonferroni tests.
(EPS)

Figure S4 **Electrophysiological characteristics of the cells in the cocultures.** Specimen responses to voltage steps in 20 mV increments from a holding potential of −64 mV (to a most negative potential of −104 mV and a most positive potential of +16 mV, for (A) an OPC with voltage-gated Na^+^ current (NaV), (B) an OPC without NaV, (C) the OPC in (A) on a faster time scale, (D) the OPC in (B) on a faster time scale, (E) a mature oligodendrocyte, (F) an interneuron, (G) a DRG neuron, (H) a satellite cell, and (I) an astrocyte. In (F and G) transient currents generated by unclamped action potentials are visible.
(EPS)

Figure S5 **Details of the signalling pathways.** (A) Mean myelination parameter of cocultures treated with NRG with TTX either added at the same time as NRG (TTX) or 3 d later (TTX 3 d). ANOVA showed significant differences across all bars (*p* = 0.025); the *p* values above bars are for comparison with NRG alone. (B–C) Western blot of control (Con) and NRG-treated pure DRG cultures (cultured as for myelinating OPC-DRG cocultures), for phosphorylated (pAkt, pERK) and total Akt and ERK, with the level of phosphorylated enzyme normalized to total enzyme in the bar charts below. The *p* values are from Student's *t* tests; numbers of experiments are shown on bars.
(EPS)

## Abbreviations

AMPA: α-Amino-3-hydroxy-5-methyl-4-isoxazolepropionic acid
AMPAR: α-Amino-3-hydroxy-5-methyl-4-isoxazolepropionic acid receptor
AP5: (2R)-amino-5-phosphonovaleric acid
BDNF: brain-derived neurotrophic factor
CNPase: 2′,3′-Cyclic-nucleotide 3′-phosphodiesterase
CNS: central nervous system
CREB: cAMP response element-binding protein
DRG: Dorsal root ganglion
Erk: extracellular-signal-regulated kinases
GFAP: Glial fibrillary acidic protein
MAPK: mitogen-activated protein kinase
MBP: myelin basic protein
MK801: [5R,10S]-[+]-5-methyl-10,11-dihydro-5H-dibenzo[a,d]cyclohepten-5,10-imine
MOG: myelin oligodendrocyte glycoprotein
NBQX: 2,3-dihydroxy-6-nitro-7-sulfamoyl-benzo[f]quinoxaline-2,3-dione
NF: neurofilament
NG2: neuron-glial antigen 2 or chondroitin sulfate proteoglycan 4
NMDA: N-methyl-D-aspartate
NMDAR: N-methyl-D-aspartate receptor
NRG: neuregulin
OPC: oligodendrocyte precursor cell
PI3K: phosphatidylinositide 3-kinases
TTX: tetrodotoxin
